# What Factors are Associated with Facilitating Conditions to Use Gerontechnology?

**DOI:** 10.1093/geroni/igab046.2496

**Published:** 2021-12-17

**Authors:** Hayoung Park, Susanna Joo, DaeEun Kim, YoonMyung Kim, Hey Jung Jun

**Affiliations:** 1 Yonsei University, Seoul, Seoul-t'ukpyolsi, Republic of Korea; 2 Yonsei University, Seodaemun-gu, Seoul-t'ukpyolsi, Republic of Korea

## Abstract

The purpose of this study is to explore relevant factors associated with facilitating conditions to use gerontechnology among Korean older adults. The sample was 310 Korean older adults aged 65 and above without cognitive impairment who participated in an online survey. The facilitating conditions to use gerontechnology were measured by the sum of five questions about basic knowledge, available help, financial resources, accessibility, and social influences of using gerontechnology from the Senior Technology Acceptance Model (STAM). Possible relevant factors comprised socio-demographic characteristics, physical and mental health, environmental factors, and social relationships. The results from the linear regression analyses showed that employment status, household income, cognitive function, social activity participation, and support from friends or neighbors were significantly associated with facilitating conditions to use gerontechnology. Older adults who are employed, have higher household income, have better cognitive functions, participate more in social activities, and receive higher levels of support from friends or neighbors tend to be in more facilitating conditions to use gerontechnology. The findings from this study imply the necessity of facilitating conditions to use gerontechnology as social policies for older adults who are unemployed, have lower household income, have worse cognitive functions, and have fewer social resources. This study is meaningful in that it has empirically explored various factors related to facilitating conditions to use gerontechnology for older adults based on the STAM. Future studies are needed to explore significant factors associated with facilitating conditions to use gerontechnology via various contexts.

